# Ficolin-2 Gene rs7851696 Polymorphism is Associated with Delayed Graft Function and Acute Rejection in Kidney Allograft Recipients

**DOI:** 10.1007/s00005-017-0475-5

**Published:** 2017-05-23

**Authors:** Ewa Dabrowska-Zamojcin, Michal Czerewaty, Damian Malinowski, Maciej Tarnowski, Sylwia Słuczanowska-Głabowska, Leszek Domanski, Krzysztof Safranow, Andrzej Pawlik

**Affiliations:** 1 Department of Experimental and Clinical Pharmacology, Pomeranian Medical University, Szczecin, Poland; 2 Department of Physiology, Pomeranian Medical University, Powstancow Wlkp. 72, 70-111 Szczecin, Poland; 3 Department of Nephrology, Transplantology and Internal Medicine, Pomeranian Medical University, Szczecin, Poland; 4 Department of Biochemistry and Medical Chemistry, Pomeranian Medical University, Szczecin, Poland

**Keywords:** Diabetes, Transplantation, Single-nucleotide polymorphism, Gene, Polymorphisms

## Abstract

Ficolin-2 is an activator of the complement system that acts via the lectin pathway. Complement activation plays a substantial role in the renal injury inherent to kidney transplantation. In this study, we examined the associations between ficolin-2 gene polymorphisms in exon 8 and kidney allograft function. This study comprised 270 Caucasian deceased-donor renal transplant recipients. The following parameters were recorded in each case: delayed graft function (DGF), acute rejection (AR), and chronic allograft dysfunction. Among patients with DGF, we observed a significantly increased frequency of rs7851696 GT and TT genotypes as well as T allele (TT + GT vs GG OR 1.98, 95% CI 1.12–3.48, *p* = 0.02; T vs G OR 2.08, 95% CI 1.27–3.41, *p* = 0.005). There was also an increased frequency of rs4521835 GG and TG genotypes as well as G alleles; however, these differences were on the borderline of statistical significance (GG + TG vs TT, OR 1.75, 95% CI 0.98–3.12, *p* = 0.07; G vs T OR 1.45, 95% CI 1.00–2.09, *p* = 0.050). In addition, we observed an increased frequency of acute allograft rejection in carriers of ficolin-2 rs7851696 T alleles on the borderline of statistical significance (TT + GT vs GG OR 1.75, 95% CI 0.97–3.16, *p* = 0.08), but the frequency of T allele was significantly higher in patients with AR (T vs G OR 1.71, 95% CI 1.02–2.87, *p* = 0.048). The results of our study suggest that ficolin-2 rs7851696 gene polymorphism influences kidney allograft functions, with T allele increasing the risk of DGF and AR.

## Introduction

Kidney transplantation is a commonly used therapy for chronic kidney diseases. Unfortunately, kidney transplantation is associated with several complications affecting graft function. Especially prevalent among these complications are delayed graft function (DGF), acute rejection (AR), and chronic allograft dysfunction (CAD). Graft transplantation induces several changes in the immune response that can lead to impaired graft function and graft loss. Both innate immunity as well as humoral and cellular immune responses are involved in these processes. The complement system plays an important role in the immune response after kidney transplantation (Salvadori and Bertoni 2016). In kidney transplantation, complement activation was found to be induced by donor brain death, renal ischemia–reperfusion injury, and AR (Ricklin et al. 2016). Activation of the complement system can be initiated via three different routes: the classical, alternative, and lectin pathways. Complement activation plays a substantial role in the renal injury inherent to kidney transplantation (Fuquay et al. 2013). Ficolin-2, a liver-synthesised protein, is the one of the lectin pathway activators of the complement system. There is growing evidence that this pathway plays a major role in the course of renal ischemia–reperfusion injury and allograft rejection (Berger et al. 2005; Renders and Heemann 2012). Ficolin-2 interacts with carbohydrate structures presented by different pathogens, generating a rapid response by activating the complement system (Petersen et al. 2001). Upon binding to distinct pathogen-associated molecular patterns, such as carbohydrates, lipoteichoic acid, and acetylated groups, ficolin-2 may facilitate phagocytosis and activation of complement through the lectin route using the same serine proteases as mannose-binding lectin (Endo et al. 2015). These observations suggest that ficolin-2 may have a role in innate immunity. The ficolin-2 gene (*FCN2*) is located on chromosome 9 (9q34), and contains eight exons and seven introns. Exon 8 encodes the C-terminal part of ficolin-2 protein and 3′UTR (Endo et al. 1996). In the ficolin-2 gene, several polymorphisms have been detected. It has been shown that a polymorphism in exon 8 (+6424 G>T, rs7851696) resulting in the amino acid substitution Ala258Ser influences the sugar-binding capacity of the protein as well as ficolin-2 gene expression (Cedzynski et al. 2007; Munthe-Fog et al. 2007). It is of interest that this allele that causes an alanine to be substituted with a serine at amino acid position 258 appears to increase the affinity of ficolin-2 towards carbohydrates (Hummelshoj et al. 2005). This polymorphism has been investigated as a risk factor for various diseases with an immune background (Ojurongbe et al. 2012; Ouf et al. 2012). The previous studies have suggested that ficolin-2 may be involved in immune responses after graft transplantation. In this study, we examined the association between ficolin-2 gene polymorphisms in exon 8 and kidney allograft function.

## Materials and Methods

This study enrolled 270 Caucasian deceased-donor renal transplant recipients (165 males, 105 females; mean age: 47.63 ± 12.96 years). The transplantation procedures were performed in the years 1999–2004. All kidneys were achieved from deceased donors. The duration of follow-up was 5 years. First renal allograft recipients were consecutively included, after giving their consent to participate in the study. Patients were excluded if they had received more than one renal transplant, if their graft had been functioning for less than 6 months, or if they failed to provide consent.

The following parameters were recorded in each case: DGF, AR, and CAD. DGF was defined as the need for dialysis during the first 7 days after transplantation. AR was diagnosed clinically and confirmed by biopsy. AR diagnoses were classified as either T-cell-mediated rejection or as mixed types. CAD was diagnosed by eliminating other causes of chronic renal dysfunction (infections, urinary obstruction, allograft artery stenosis, or cyclosporine toxicity) and by changes in biopsy samples. The process was diagnosed clinically in patients having a slow persistent rise in serum creatinine at least 30% above baseline, usually accompanied by new or worsening hypertension and proteinuria (above 500 mg/24 h). Biopsy criteria included the presence of interstitial fibrosis, tubular atrophy, hypertrophy of the arterial intima and smooth muscle (intimal thickening), and glomerular sclerosis. All biopsies were reviewed by a renal pathologist using the Banff working classification criteria (Solez et al. 2008). All patients received a standard immunosuppressive protocol with triple drug therapy including a calcineurin inhibitor (cyclosporine A in 75% of patients and tacrolimus in 24%), azathioprine (55%) or mycophenolate mofetil (37%), and steroids (91%). Informed consent was obtained from all patients. The local ethics committee of the Pomeranian Medical University in Szczecin, Poland, approved the study protocol.

## Methods

### FCN2 Genotyping

DNA was isolated from peripheral blood using the Genomic Mini AX Blood 1000 Spin kit (A&A Biotechnology, Gdańsk, Poland). A 384 bp DNA fragment from exon 8 of the ficolin-2 gene was obtained by PCR amplification, using the primers 5′-CTGTCTGTAATGATGTTACTGC-3′ and 5′-TACAAACCGTAGGGCCAAGC-3′ (Wu et al. 2014). The cycling conditions were 94 °C for 4 min, 35 cycles of 94 °C for 30 s, 56 °C for 30 s, 72 °C for 30 s, and finally 72 °C for 5 min. The PCR products were tested by agarose gel electrophoresis and then subjected to DNA sequencing on an ABI 3130 Genetic Analyzer (Applied Biosystems, CA, USA). In total, this DNA fragment contains 86 single-nucleotide polymorphisms (SNPs) according to the NCBI dbSNP database. Only three of them (rs7851696, rs17549193, and rs4521835) were polymorphic in our samples and only these SNPs were further analysed. In addition, all samples were genotyped in duplicate using allelic discrimination with the TaqMan^®^ predesigned SNP Genotyping Assay, including appropriate primers and fluorescently labelled (FAM and VIC) MGB™ probes to detect the alleles of *ficolin*-*2* rs7851696 (assay ID: C__29220549_20). TaqMan^®^ probes (Applied Biosystems, CA, USA) were used and the procedure was carried out using a ViiA™ 7 Real-time PCR system (Applied Biosystems, CA, USA).

### Statistical Analysis

The consistency of the genotype distribution with the Hardy–Weinberg equilibrium was assessed using exact test. A *χ* ^2^-square test and Fisher’s exact test were used to compare genotype and allele distributions between the groups. The number of acute rejection episodes and creatinine concentrations was compared between genotype groups using the Mann–Whitney test. Cox proportional hazards model was used to calculate hazard ratio (HR) for associations between the genotypes and permanent graft loss. A multivariate logistic regression model was used to find independent predictors of DGF and acute rejection risk. *p* values <0.05 without correction for multiple comparisons were considered statistically significant.

## Results

The distribution of the ficolin-2 rs17549193, rs4521835, and rs7851696 genotypes was in Hardy–Weinberg equilibrium (*p* = 0.77, *p* = 0.14, and *p* = 0.80, respectively). Among patients with DGF, we observed a statistically significantly increased frequency of rs7851696 GT and TT genotypes as well as T alleles (TT + GT vs GG, OR 1.98, 95% CI 1.12–3.48, *p* = 0.02; T vs G, OR 2.08, 95% CI 1.27–3.41, *p* = 0.005) (Table 1), as well as an increased frequency of rs4521835 GG and TG genotypes as well as G alleles, although these differences were on the borderline of statistical significance (GG + TG vs TT, OR 1.75, 95% CI 0.98–3.12, *p* = 0.07; G vs T, OR 1.45, 95% CI 1.00–2.09, *p* = 0.05).

**Table 1:** Association between *FCN2* rs17549193, rs7851696, and rs4521835 genotypes, and delayed graft function (DGF)

	DGF		Without DGF		*p* ^a^	Comparison	*p* ^b^	OR (95% CI)
	*n*	%	*n*	%				
*FCN2* rs17549193								
Genotype								
CC	38	44.71	94	51.09	0.62	TT + CT vs CC	0.36	1.29 (0.77–2.16)
CT	38	44.71	73	39.67		TT vs CT + CC	0.83	1.16 (0.50–2.73)
TT	9	10.58	17	9.24		TT vs CC	0.64	1.31 (0.54–3.19)
						CT vs CC	0.41	1.29 (0.75–2.22)
						TT vs CT	1.00	1.02 (0.41–2.50)
Allele								
C	114	67.06	261	70.92				
T	56	32.94	107	29.08		T vs C	0.37	1.20 (0.81–1.77)
*FCN2* rs7851696								
Genotype								
GG	55	64.71	145	78.38	0.005*	TT + GT vs GG	0.02*	1.98 (1.12–3.48)
GT	25	29.41	39	21.08		TT vs GT + GG	0.013*	11.50 (1.32–100.02)
TT	5	5.88	1	0.54		TT vs GG	0.009*	13.18 (1.51–115.38)
						GT vs GG	0.09	1.69 (0.94–3.05)
						TT vs GT	0.08	7.80 (0.86–70.75)
Allele								
G	135	79.41	329	88.92				
T	35	20.59	41	11.08		T vs G	0.005*	2.08 (1.27–3.41)
*FCN2* rs4521835								
Genotype								
TT	21	24.71	66	36.46	0.14	GG + TG vs TT	0.07	1.75 (0.98–3.12)
TG	41	48.23	78	43.10		GG vs TG + TT	0.27	1.44 (0.79–2.63)
GG	23	27.06	37	20.44		GG vs TT	0.07	1.95 (0.96–4.00)
						TG vs TT	0.13	1.65 (0.89–3.07)
						GG vs TG	0.62	1.18 (0.62–2.25)
Allele								
T	83	48.82	210	58.01				
G	87	51.18	152	41.99		G vs T	0.050	1.45 (1.00–2.09)

*FCN2* ficolin-2 gene, *OR* odds ration, *95% CI* 95% confidence interval

* *p* < 0.05

^a^ *χ* ^2^ test

^b^Fisher exact test

In addition, we observed an increased frequency of acute allograft rejection in carriers of ficolin-2 rs7851696 on the borderline of statistical significance (TT + GT vs GG, OR 1.75, 95% CI 0.97–3.16, *p* = 0.08), but the frequency of T allele was significantly higher in patients with AR (T vs G, OR 1.71, 95% CI 1.02–2.87, *p* = 0.048) (Table 2). Moreover, GT and TT genotypes were associated with an increased number of acute rejection episodes (GT + TT vs GG, *p* = 0.038) (Table 3).

**Table 2:** Association between *FCN2* rs17549193, rs7851696, and rs4521835 genotypes, and acute rejection (AR)

	AR		Without AR		*p* ^a^		*p* ^b^	OR (95% CI)
	*n*	%	*n*	%				
*FCN2* rs17549193								
Genotype								
CC	34	48.57	98	49.25	0.84	TT + CT vs CC	1.00	1.03 (0.60–1.77)
CT	28	40.00	83	41.71		TT vs CT + CC	0.64	1.30 (0.54–3.13)
TT	8	11.43	18	9.04		TT vs CC	0.63	1.28 (0.51–3.21)
						CT vs CC	1.00	0.97 (0.54–1.74)
						TT vs CT	0.62	1.32 (0.52–3.36)
Allele								
C	96	68.57	279	70.10				
T	44	31.43	119	29.90		T vs C	0.75	1.08 (0.71–1.63)
*FCN2* rs7851696								
Genotype								
GG	46	65.71	154	77.00	0.12	TT + GT vs GG	0.08	1.75 (0.97–3.16)
GT	21	30.00	43	21.50		TT vs GT + GG	0.18	2.94 (0.58–14.92)
TT	3	4.29	3	1.50		TT vs GG	0.15	3.35 (0.65–17.15)
						GT vs GG	0.14	1.64 (0.88–3.03)
						TT vs GT	0.41	2.05 (0.38–11.02)
Allele								
G	113	80.71	351	87.75				
T	27	19.29	49	12.25		T vs G	0.048	1.71 (1.02–2.87)
*FCN2* rs4521835								
Genotype								
TT	17	24.64	70	35.53	0.22	GG + TG vs TT	0.10	1.69 (0.91–3.14)
TG	36	52.17	83	42.13		GG vs TG + TT	0.87	1.05 (0.55–2.01)
GG	16	23.19	44	22.34		GG vs TT	0.32	1.50 (0.69–3.27)
						TG vs TT	0.11	1.79 (0.92–3.45)
						GG vs TG	0.73	0.84 (0.42–1.68)
Allele								
T	70	50.72	223	56.60				
G	68	49.28	171	43.40		G vs T	0.24	1.27 (0.86–1.87)

*FCN2* ficolin-2 gene, *OR* odds ratio, *95% CI* 95% confidence interval

^a^ *χ* ^2^ test

^b^Fisher exact test

**Table 3:** Association between *FCN2* rs17549193, rs7851696, and rs4521835 genotypes, and the number of episodes of acute rejection per patient

Genotype	CC		CT		TT		CT + TT vs CC	TT vs CC + CT
	*n*	Mean ± SD	*n*	Mean ± SD	*n*	Mean ± SD	*p*	*p*
*FCN2* rs17549193	42	0.32 ± 0.62	32	0.29 ± 0.53	8	0.31 ± 0.47	0.97	0.65

Genotype	GG		GT		TT		GT + TT vs GG	TT vs GG + GT
	*n*	Mean ± SD	*n*	Mean ± SD	*n*	Mean ± SD	*p*	*p*
*FCN2* rs7851696	50	0.25 ± 0.49	27	0.42 ± 0.69	5	0.83 ± 1.17	0.038*	0.13

Genotype	TT		TG		GG		TG + GG vs TT	GG vs TT + TG
	*n*	Mean ± SD	*n*	Mean ± SD	*n*	Mean ± SD	*p*	*p*
*FCN2* rs4521835	19	0.22 ± 0.49	41	0.35 ± 0.57	21	0.35 ± 0.66	0.083	0.76

*p* values were calculated with the Mann–Whitney test

*SD* standard deviation, *FCN2* ficolin-2 gene, *n* – total number of acute rejection episodes in patients with given genotype

* *p* < 0.05

There were no statistically significant associations between ficolin-2 gene polymorphisms and CAD (Table 4) or with serum creatinine concentrations 1–60 months after transplantation, with the exception of increased creatinine levels after 3 and 6 months after transplantation in carriers of the rs4521835 GG and GT genotypes [in comparison with patients with the TT genotype (Table 5)]. There were no significant associations between the number of minor alleles for each SNP and the risk of permanent graft loss in Cox proportional hazards model (HR 1.20, 95% CI 0.65–2.21, *p* = 0.56 for rs17549193 allele T; HR 1.65, 95% CI 0.79–3.45, *p* = 0.19 for rs7851696 allele T; HR 1.59, 95% CI 0.90–2.80, *p* = 0.11 for rs4521835 allele G).

**Table 4:** Association between *FCN2* rs17549193, rs7851696, and rs4521835 genotypes, and chronic allograft dysfunction (CAD)

	CAD		Without CAD		*P* ^a^		*p* ^b^	OR (95% CI)
	*n*	%	*n*	%				
*FCN2* rs17549193								
Genotype								
CC	31	50.00	101	48.79	0.54	TT + CT vs CC	0.89	0.95 (0.54–1.68)
CT	23	37.10	88	42.51		TT vs CT + CC	0.33	1.56 (0.64–3.77)
TT	8	12.90	18	8.70		TT vs CC	0.46	1.45 (0.57–3.65)
						CT vs CC	0.64	0.85 (0.46–1.57)
						TT vs CT	0.30	1.70 (0.66–4.40)
Allele								
C	85	68.55	290	70.05				
T	39	31.45	124	29.95		T vs C	0.74	1.07 (0.70–1.66)
*FCN2* rs7851696								
Genotype								
GG	41	66.13	159	76.44	0.19	TT + GT vs GG	0.14	1.66 (0.90–3.08)
GT	20	32.26	44	21.16		TT vs GT + GG	1.00	0.67 (0.08–5.81)
TT	1	1.61	5	2.40		TT vs GG	1.00	0.78 (0.09–6.82)
						GT vs GG	0.09	1.76 (0.94–3.31)
						TT vs GT	0.66	0.44 (0.05–4.02)
Allele								
G	102	82.26	362	87.02				
T	22	17.74	54	12.98		T vs G	0.19	1.45 (0.84–2.49)
*FCN2* rs4521835								
Genotype								
TT	18	29.03	69	33.82	0.70	GG + TG vs TT	0.54	1.25 (0.67–2.32)
TG	28	45.16	91	44.61		GG vs TG + TT	0.49	1.27 (0.65–2.45)
GG	16	25.81	44	21.57		GG vs TT	0.43	1.39 (0.64–3.02)
						TG vs TT	0.74	1.18 (0.60–2.30)
						GG vs TG	0.71	1.18 (0.58–2.41)
Allele								
T	64	51.61	229	56.13				
G	60	48.39	179	43.87		G vs T	0.41	1.20 (0.80–1.79)

*FCN2* ficolin-2 gene, OR odds ratio, *95% CI* 95% confidence interval

^a^ *χ* ^2^ test

^b^Fisher exact test

**Table 5:** Serum creatinine concentrations after transplantation in renal graft recipients stratified according to the *FCN2* rs17549193 C>T, *FCN2* rs4521835 T>G, and *FCN2* rs7851696 G>T gene polymorphism genotypes

Time after Tx (month)	rs17549193:C>T *FCN2* genotype				rs4521835:T>G *FCN2* genotype				rs7851696:G > T *FCN2* genotype			
	CC	CT	TT	TT + CT vs CC	TT	TG	GG	GG + TG vs TT	GG	GT	TT	TT + GT vs GG
	Mean ± SD	Mean ± SD	Mean ± SD	*p*	Mean ± SD	Mean ± SD	Mean ± SD	*p*	Mean ± SD	Mean ± SD	Mean ± SD	*p*
Creatinine (mg/dL)												
1	1.84 ± 0.95	1.85 ± 0.71	1.79 ± 0.56	0.11	1.76 ± 0.81	1.91 ± 0.75	1.81 ± 0.99	0.06	1.80 ± 0.70	1.78 ± 0.82	3.38 ± 2.39	0.82
3	1.72 ± 0.60	1.78 ± 0.58	1.81 ± 0.59	0.12	1.65 ± 0.55	1.88 ± 0.63	1.68 ± 0.54	0.04*	1.74 ± 0.56	1.77 ± 0.65	2.02 ± 0.81	0.85
6	1.72 ± 0.66	1.80 ± 0.57	1.81 ± 0.60	0.07	1.66 ± 0.53	1.89 ± 0.70	1.68 ± 0.52	0.03*	1.75 ± 0.58	1.71 ± 0.52	2.52 ± 1.78	0.96
12	1.69 ± 0.56	1.82 ± 0.64	1.73 ± 0.54	0.08	1.66 ± 0.51	1.87 ± 0.65	1.67 ± 0.55	0.08	1.75 ± 0.59	1.67 ± 0.56	2.32 ± 0.89	0.65
24	1.73 ± 0.60	1.71 ± 0.49	1.79 ± 0.56	0.65	1.70 ± 0.58	1.79 ± 0.54	1.67 ± 0.53	0.36	1.74 ± 0.55	1.65 ± 0.58	2.09 ± 0.42	0.38
36	1.73 ± 0.66	1.70 ± 0.52	1.76 ± 0.54	0.66	1.69 ± 0.64	1.76 ± 0.57	1.69 ± 0.57	0.25	1.72 ± 0.60	1.66 ± 0.55	2.31 ± 0.85	0.98
48	1.73 ± 0.68	1.80 ± 0.60	1.73 ± 0.53	0.19	1.67 ± 0.62	1.85 ± 0.65	1.71 ± 0.63	0.08	1.75 ± 0.61	1.79 ± 0.72	1.76 ± 0.54	0.90
60	1.74 ± 0.73	1.69 ± 0.55	1.77 ± 0.57	0.56	1.76 ± 0.74	1.75 ± 0.64	1.59 ± 0.47	0.97	1.74 ± 0.65	1.64 ± 0.64	1.80 ± 0.62	0.37

*p* value was calculated with the Mann–Whitney test

*SD* standard deviation, *FCN2* ficolin-2 gene, *OR* odds ratio; 9*5% CI* 95% confidence interval

* *p* < 0.05

In the multivariate regression analysis, after taking into the account graft recipients’ sex, age, and number of ficolin-2 rs7851696 T alleles, we analysed the factors predisposing to DGF and acute allograft rejection. In this analysis, the number of T alleles was positively associated with DGF (*p* = 0.004) (Table 6). The similar positive association with acute allograft rejection was on the borderline of statistical significance (*p* = 0.051) (Table 6).

**Table 6:** Multivariate logistic regression analysis with delayed graft function (DGF) and acute rejection as the dependent variable

Independent variables	DGF		Acute rejection	
	OR (95% CI)	*p*	OR (95% CI)	*p*
Sex (male vs female)	1.52 (0.86–2.66)	0.15	1.46 (0.81–2.64)	0.21
Age (years)	1.01 (0.98–1.03)	0.67	0.98 (0.95–1.00)	0.029
*FCN2* rs7851696 (number of T alleles)	2.10 (1.26–3.48)	0.004	1.68 (1.00–2.85)	0.051

*FCN2* ficolin-2 gene, *OR* odds ratio, *95% CI* 95% confidence interval

## Discussion

In this study, we examined the association between ficolin-2 gene polymorphisms and kidney allograft function. These polymorphisms were studied in kidney allograft recipients; all kidneys were achieved from deceased donors. The results of this study suggest that the ficolin-2 rs7851696 T allele is associated with an increased risk of DGF (*p* = 0.005) as well as acute kidney allograft rejection (*p* = 0.048). The previous studies have shown that the ficolin-2 rs7851696 T allele is associated with increased affinity of lectin-2 for carbohydrate structures presented by different pathogens (Hummelshoj et al. 2005). It results in enhanced activation of complement, which plays a significant role in immune processes influencing allograft function. The previous studies have shown that kidney graft recipients who experienced DGF showed an increased risk of acute rejection and long-term graft failure (Moore et al. 2008; Nicoletto et al. 2014). Rejection is the major clinical problem accounting for most graft failures. Delayed graft function is recognised when the patient needs dialysis in first 7 days after renal transplantation. Ischemic damage is the most common cause of DGF, but tissue inflammation can also lead to renal ischemia and to DGF. Delayed graft function is characterised by tubular dysfunction, interstitial inflammation, and altered microcirculation (Moore et al. 2008; Ponticelli 2014). Several studies have shown that complement may be involved in the pathogenesis of DGF (Castellano et al. 2016; Damman et al. 2015; Pushpakumar et al. 2011; Yu et al. 2016). Yu et al. (2016) have shown that blocking the terminal complement pathway prevented reperfusion injury and increased renal graft survival. These data suggest that complement inhibitors can prevent the development of DGF. Pushpakumar et al. (2011) suggested that complement control at the endothelial barrier modulates complement function during the first hours after kidney transplantation and that blocking of the C3 component reduced the risk of ischemia–reperfusion injury. Castellano et al. (2016) suggest that complement might be pivotal in the down-regulation of Klotho in reperfusion injury of kidney grafts. Klotho is an anti-aging factor mainly produced by renal tubular epithelial cells, and is down-regulated in acute kidney injury. Acquired deficiency of Klotho, after activation of complement, might contribute to DGF-associated CAD. In other studies, Castellano et al. (2010) have shown that C1-inhibitor administration leads to significant inhibition of tubular damage, and have suggested that inhibition of the classical and lectin pathways in complement activation may represent a novel therapeutic approach for the prevention of DGF in kidney graft recipients. Pratt et al. (2002) have described complement as a significant part of innate immunity, which is recognised as a contributor to inflammation in transplant rejection. These authors have shown that the C3 component can enhance the process of renal allograft rejection. These results indicate that improved success in kidney transplantation could be achieved by therapeutic manipulation of innate immunity involving blocking activation pathways of the complement system.

Genetic polymorphisms of ficolin-2 in the donors and the recipients may be responsible for different responses toward foreign antigens or renal injury and thus may be involved in the process of graft damage. Eikmans et al. (2012) have shown that donor ficolin-2 gene polymorphism in rs7851696 was associated with risk of acute allograft rejection. Messenger RNA expression of ficolin-2 was detected in donor kidney and also in peripheral blood mononuclear cells, monocytes, and differentiated macrophages (Eikmans et al. 2012). The opposite result was obtained by Damman et al. (2012) who suggest that ficolin-2 gene polymorphisms of the donor and recipient do not influence graft outcome after kidney transplantation. The results of Wu et al. (2014) also suggest a lack of association between recipient ficolin-2 gene polymorphisms and risk of acute kidney allograft rejection. Other studies have shown that ficolin-2 rs7851696 variants have a significant impact on the risk of developing bloodstream infections after kidney transplantation, due to the decreased binding capacity of ficolin-2 towards *N*-acetyl glucosamine on microbial surfaces (Wan et al. 2013). This polymorphism was also associated with a predisposition to bacterial and cytomegalovirus infection after liver transplantation (de Rooij et al. 2010, 2011).

The limitation of our study is that we found associations which were significant only without correction for multiple comparisons. Neither of the associations would remain significant if Bonferroni correction was used.

The results of our study suggest that ficolin-2 rs7851696 gene polymorphisms influence kidney allograft functions, increasing the risk of DGF and AR. However, this hypothesis requires further investigation.
